# Crewkerne Hospital—An Explanation

**Published:** 1910-08-13

**Authors:** John Dyson

**Affiliations:** Chairman of House Committee. Moorlands, near Crewkerne, Somerset


### CREWKERNE HOSPITAL—AN EXPLANATION.

*To the Editor of* The Hospital.

Sir,—Owing to long absence from home, I did not see until the other day the report (No. LIV.) on Crewkerne Hospital in your issue of June 25. The matron's bedroom was, it is true, intended for a pay-ward, but was never so used, a room on the ground floor having been substituted from the opening of the hospital in 1904 (not, as stated in the report, 1894). When Sir Henry Burdett paid his visit the matron was away, and her place was taken by a nurse from Taunton, who was imperfectly acquainted with the facts.

I shall be much obliged by your inserting this explanation.—Yours faithfully,

John Dyson,
Chairman of House Committee.

Moorlands,
near Crewkerne, Somerset,
August 4, 1910.

